# Comparison of glyburide and insulin in the management of gestational diabetes: A meta-analysis

**DOI:** 10.1371/journal.pone.0182488

**Published:** 2017-08-03

**Authors:** Rongjing Song, Ling Chen, Yue Chen, Xia Si, Yi Liu, Yue Liu, David M. Irwin, Wanyu Feng

**Affiliations:** 1 Department of Pharmacy, Peking University People’s Hospital, Beijing, China; 2 Department of Endocrinology and Metabolism, Peking University People's Hospital, Beijing, China; 3 Department of Pharmacology, Peking University, Health Science Center, Beijing, China; 4 Department of Laboratory Medicine and Pathobiology, University of Toronto, Toronto, Canada; Shanghai Jiaotong University School of Medicine Xinhua Hospital, CHINA

## Abstract

**Objective:**

The aim of this meta-analysis was to determine the efficacy and safety of glyburide as a treatment for gestational diabetes mellitus (GDM) compared to insulin.

**Methods:**

A meta-analysis was conducted to compare the management of gestational diabetes with glyburide and insulin. Studies fulfilling all of the following inclusion criteria were included in this meta-analysis: subjects were women with gestational diabetes requiring drug treatment; the comparison treatment included glyburide vs insulin; one or more outcomes (maternal or neonatal) should be provided in the individual study; the study design should be a randomized control trial. Exclusion criteria: non-RCT studies; non-human data. PubMed, Embase and CENTRAL databases were searched from inception until 10 October 2016.

**Results:**

Ten randomized control trials involving 1194 participants met the inclusion criteria and were included. 13 primary outcomes (6 maternal, 7 neonatal) and 26 secondary outcomes (9 maternal, 17 neonatal) were detected and analyzed in this study. Glyburide significantly increased the risk of any neonatal hypoglycemia [risk ratio (RR), 1.89; 95% confidence interval (95%CI), 1.26 to 2.82; p = 0.002]. Sensitivity analysis confirmed robustness of this result [RR, 2.29; 95%CI, 1.49 to 3.54; p = 0.0002]. No differences were observed between the two groups with respect to birth weights [mean difference (MD), 79; 95%CI, -64 to 221.99; p = 0.28] and the risk of macrosomia [RR, 1.69; 95%CI, 0.57 to 5.08; p = 0.35].

**Conclusion:**

For women with gestational diabetes, no differences in maternal short term outcomes were observed in those treated with glyburide or insulin. However, the incidence of neonatal hypoglycemia was higher in the glyburide group compared to the insulin group.

## Introduction

Gestational diabetes mellitus (GDM) was originally defined as any degree of glucose intolerance that was first recognized during pregnancy [1]. More recently, the American Diabetes Association has recommended that women diagnosed with diabetes in the first trimester be classified as having type 2 diabetes, whereas GDM should be defined as diabetes diagnosed in the second or third trimester of pregnancy that is clearly neither type 1 nor type 2 diabetes [2]. According to the “International Association of Diabetes and Pregnancy Study Group (IADPSG) diagnostic criteria” [3], the prevalence of GDM has been reported to be 17.6%, 4.2%, 11.8%, 9.5%, 23.3%, 8.6%, and 45% in Singapore [4], Greenland [5], Switzerland [6], South Korea [7], Sri Lanka [8], Sub-Saharan Africa [9], and the United Arab Emirates [10], respectively. In recent years, the prevalence of GDM has increased, which has been attributed to the higher incidence of obesity in the general population and the increase in the number of pregnancies in older women [11]. GDM carries risks for both the mother and the infant [12]. In mothers, it is associated with a higher risk of pregnancy-induced hypertension, pre-eclampsia, cesarean delivery, and an increased risk of developing diabetes later in life. Infants of women with GDM are at a higher risk of neonatal hypoglycemia, macrosomia, respiratory distress syndrome, neonatal death, and stillbirth [13]. Therefore, GDM is associated with significant transgenerational maternal and neonatal morbidity [14].

Glucose levels can be managed by lifestyle changes alone in most patients with GDM. Over the past few decades, insulin therapy has been the first-line agent recommended for the treatment of GDM in patients that have failed to achieve desired glycemic goals through lifestyle changes [15, 16]. However, there are several disadvantages to this approach, including hypoglycemia, weight gain, the requirement for multiple daily subcutaneous injections, the need to train patients in the required technique, and an increased medical cost burden [17, 18]. In recent years, a growing body of research has suggested that oral hypoglycemic agents, such as glyburide and metformin, could be used for the treatment of GDM [15, 19–24]. Metformin has been increasingly recognized as an alternative to insulin therapy for GDM [16, 25] and there is strong evidence for its effectiveness and safety [26, 27]. Currently, treatment with metformin is preferable to insulin for maternal health if it sufficiently controls hyperglycemia [2, 11].

Glyburide belongs to the class of longer-acting sulfonylureas. Data regarding its use in GDM are conflicting [18, 24, 26–31]. Two new randomized control trials comparing treatment with glyburide and insulin in GDM have recently been published [29, 32]. A meta-analysis of the updated data, including previously available data and the recently published trials, might provide stronger evidence with respect to the effectiveness and safety of glyburide. Therefore, the aim of our study was to reassess the efficacy and safety of glyburide compared to those of insulin in the management of GDM based on all available data. These findings will provide valuable evidence regarding the use of glyburide in the treatment of GDM.

## Materials and methods

### Inclusion and exclusion criteria

Inclusion criteria: study subjects were GDM patients who were not well controlled with lifestyle adjustment and needed drug treatment to control their glycemic levels; the treatment schedule in the control and interventional group were insulin and glyburide, respectively; the study design was randomized control trial; primary or secondary outcomes reported in the trials included maternal weight gain during pregnancy, type of delivery, neonatal hypoglycemia, birth weight, and macrosomia; the language used for the individual studies was not limited; only studies with full text available online were included in the study. Studies meeting all the above criteria were included in the meta-analysis.

Exclusion criteria: retrospective cohort study; case report; reviews; animal experiment.

### Literature search and study selection

Databases searched included PubMed, EMBASE, and CENTRAL (Cochrane Cental Register of Controlled Trials), from inception through October 2016. The following keywords were used in the searches: glyburide or glibenclamide; gestational diabetes, gestational diabetes mellitus; randomized controlled trials. S1 Table shows the detailed search strategy. Additionally, literature including unpublished studies, data from academic conference and dissertations were manually identified in searches from Google Scholar and other sources. Two investigators performed the search processes and study selection independently according to the above criteria. Discrepancies were resolved by discussion or by involving a third assessor.

### Assessment of risk of bias and data collection

Two review authors independently assessed the quality of each included study by using the tool in the Cochrane Handbook for Systematic Reviews of Intervention. The major biases including random sequence generation, allocation concealment, blinding of participants and personnel, blinding of outcome assessment, incomplete outcome data, selective reporting, and other biases. Low risk, high risk or unclear risk was used in the assessment. The overall risk of bias was presented in six domains. Data were collected for baseline information and outcomes according to the above criteria. Any disagreement was resolved by discussion.

### Statistical analysis

Review Manager software (version 5.3) was used to perform the statistical analysis. Data were pooled according to the type of outcomes. Dichotomous outcomes were calculated using risk ratio (RR) with 95% confidence intervals (CI). Continuous outcomes were calculated using mean difference (MD) with 95% confidence intervals (CI). The Inverse Variance method was used for continuous outcomes and the Mantel Haenszel method for dichotomous outcomes. Heterogeneity was measured by I^2^ statistics (I^2^>50% was regarded as heterogeneity) and Q statistics (P<0.1 was considered heterogeneity). A fixed-effects model was used when I^2^<50% (homogeneity), otherwise a random-effects model was used. P values less than 0.05 were considered to be statistically significant. Potential publication bias was assessed by Funnel plots.

## Results

### Literature search and study characteristics

**Fig 1:**
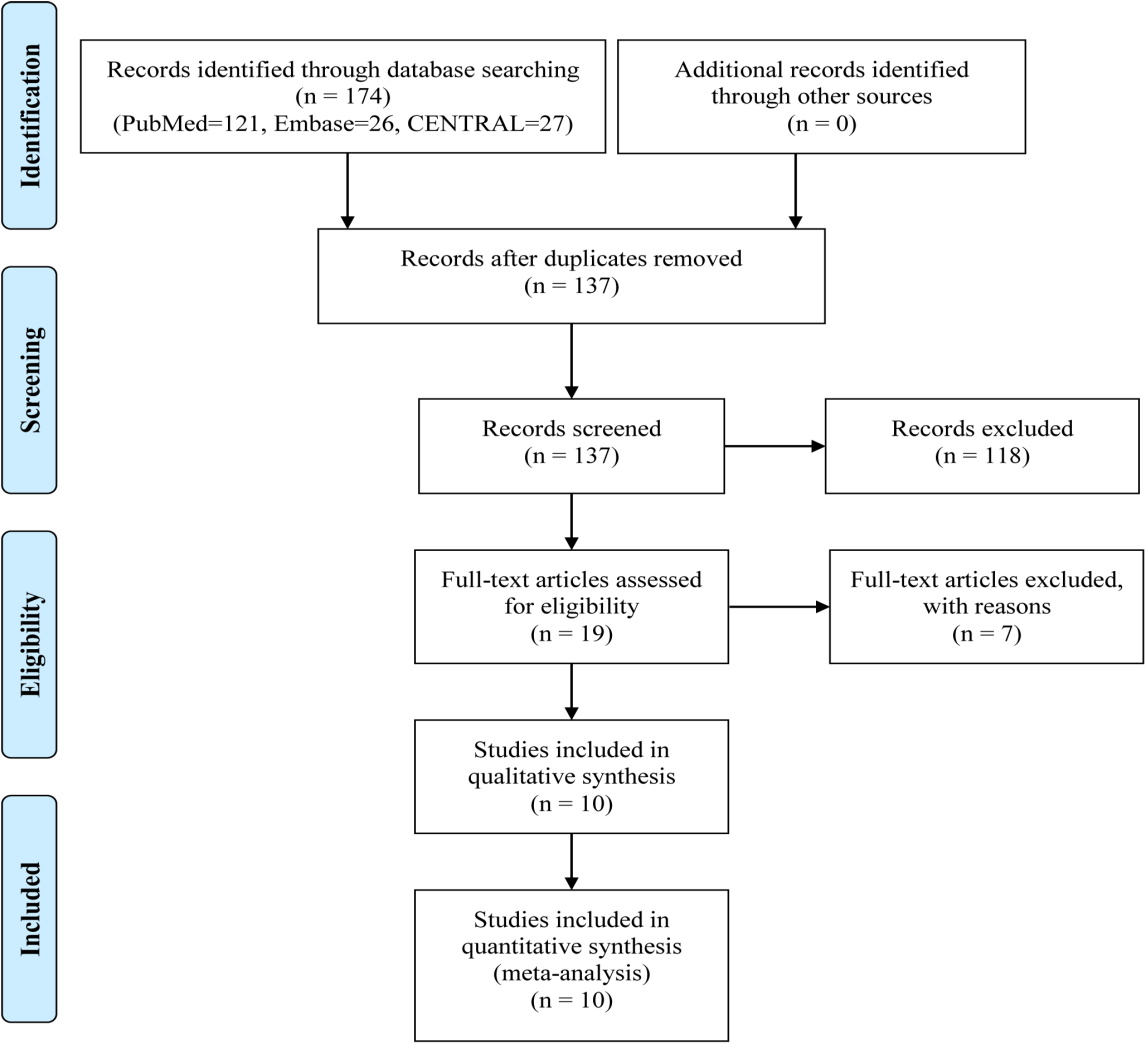
Study selection flowchart.

S1 Table provides the detailed search strategy. A total of 174 articles were found through the searches of three databases (PubMed, EMBASE, and CENTRAL), with 137 records left after removing duplicates. After reading the titles or abstracts, 118 were excluded leaving 19 studies for further review. Fig 1 shows the process of the literature search and selection. A final set of ten randomized control trials with a total of 1194 participants meeting the inclusion criteria were included in this meta-analysis (575 on glyburide; 619 on insulin) [19, 24, 28, 29, 32–37]. Countries included in these studies were USA, Brazil, India and Iran. The two larger studies involved more than 100 subjects each [19, 32], while the other eight trials involved fewer than 100 participants each. Characteristics and baseline data are shown in Tables 1–3, respectively. Baseline data were collected and compared for maternal age, prepregancy BMI (body mess index), gestational age at entry, fasting plasma glucose at OGTT (oral glucose tolerance test), 2h postprandial glucose at OGTT, and HbA1c (glycated haemoglobin) level at entry (see Table 4), and the results indicated that there was no difference observed between the groups for these baseline characteristics.

**Table 1:** Characteristics of the studies included in the meta-analysis.

Author/year	Country	Study period	Participants	Criteria for GDM diagnosis	Criteria for starting drug treatment (mmol/l)	No. of outcomes
**Langer** **2000**	USA	NA	Singleton pregnancies	100 g OGTT; 2 or more abnormal; F≥5.3mmol/L; 1h≥10mmol/L; 2h≥8.6mmol/L; 3h≥7.8mmol/L	F≥5.3 2h≥6.7	21
**Bertini** **2005**	Brazil	October 1, 2003 to July 1, 2004	Gestational age 11–33 weeks with single gestations	75 g OGTT; Any abnormal; F≥6.1mmol/L; 2h≥7.8mmol/L	NA	10
**Silva** **2007**	Brazil	October 1, 2003 to March 8, 2005	Gestational age 11–33 weeks, single fetus without malformation, absence of other pathologies	75 g OGTT; Any abnormal; F≥6.1mmol/L; 2h≥7.8mmol/L	F≥5.0 2h≥5.6	7
**Anjalakshi** **2007**	India	NA	Singleton pregnancies	75 g OGTT; 2 h>7.8mmol/L	2h≥6.7	5
**Ogunyemi** **2007**	USA	2002 to 2005	NA	NA	NA	7
**Lain** **2009**	USA	2002 to 2005	Gestational age 24–34 weeks; singleton pregnancies; no known fetal anomalies or intrauterine growth retardation; no use of other medications with known glycemic effect	100 g OGTT; 2 or more abnormal; F≥5.3mmol/L; 1h≥10mmol/L; 2h≥8.6mmol/L; 3h≥7.8mmol/L	F>5.3 2h>6.7	46
**Mukhopadhyay** **2012**	India	January 1, 2010 to December 31, 2010	Gestational age 20–28 weeks with singleton pregnancies	75 g OGTT; 2 h>7.8mmol/L	F≈5.0 2h≈6.7	8
**Tempe** **2013**	India	December 2008 to December 2009	Gestational diabetes not responding to diet control; singleton pregnancies; normal liver and kidney function tests; regular antenatal clinic visits	100 g OGTT; 2 or more abnormal; F≥5.3mmol/L; 1h≥10mmol/L; 2h≥8.6mmol/L; 3h≥7.8mmol/L	F>5.3 2h>6.7	14
**Mirzamoradi 2015**	Iran	March 2012 to March 2013	Women aged 18–45 years with singleton pregnancies and in their 24–36 weeks	F>5.3mmol/L, 1h>10mmol/L or 2h>8.3mmol/L	F≥5.0 2h≥6.7	14
**Behrashi** **2016**	Iran	NA	Women aged 18–45 years with singleton pregnancies and in their 11–33 weeks, absence of diabetes before pregnancy, absence of known kidney, hepatic, hematological, and/or cardiovascular disease	100 g OGTT; 2 or more abnormal; F≥5.3mmol/L; 1h≥10mmol/L; 2h≥8.6mmol/L; 3h≥7.8mmol/L	F≥5.0 2h≥6.7	11

Abbreviations are as follows: NA, not available; F, fasting; 1h, 1h postprandial; 2h, 2h postprandial; 3h, 3h postprandial.

**Table 2:** Baseline characteristics of studies comparing glyburide and insulin in the treatment of GDM.

Author/year	No.of patients (Gly/Ins)	Age (years) (Gly/Ins)	GA at entry (weeks) (Gly/Ins)	Prepregnancy BMI(Kg/m2) (Gly/Ins)	OGTT fasting- 2h blood glucose (mmol/l) (Gly/Ins)	HbA1c at entry(%) (Gly/Ins)	Glyburide	Insulin	The type of insulin
**Langer** **2000**	201/203	29.0/30.0	28.0/27.0	NA	5.43–9.74/5.49–9.74	5.7/5.6	Starting dose: 2.5 mg/day; Dose titration: 2.5 mg increase during initial week, thereafter 5 mg/week as necessary; Maximum dose: 20 mg/day	Starting dose: 0.7U/kg, 3 times daily; Increased weekly as necessary	NA
**Bertini** **2005**	24/27	31.2/28.7	NA	27.5/27.0	No statistical difference	NA	Starting dose: 5 mg/day; Dose titration: dose increased per week as necessary; Maximum dose: 20 mg/day	0.7 U/kg in the first trimester; 0.8 U/kg in the second trimester; 0.9 U/kg in the third trimester	Regular insulin and NPH
**Silva** **2007**	32/36	31.6/29.9	26.6/25.6	27.5/27.9	NA	NA	Starting dose: 2.5 mg/day; Dose titration: 2.5 mg increase per week as necessary; Maximum dose: 20 mg/day	0.7 U/kg in the 1st quarter; 0.8 U/kg in the second quarter; 0.9 U/kg in the third quarter	Regular insulin and NPH
**Anjalakshi** **2007**	10/13	24.9/27.5	22.5/22.6	22.8/25.3	NA	5.48/5.75	Starting dose: 0.625 mg/day; Dose titration: once a week to maintain 2h PG≤6.7 mmol/L	Starting dose: 0.1U/kg; Increased weekly as necessary.	NA
**Ogunyemi** **2007**	48/49	Non significant	28.1/24.6	32.0/30.8	5.76–9.94/6.43–11.0	5.8/7.5	Not reported; (Mean final dose: 5 mg/day)	Not reported (Mean final dose: NPH 30 units and regular 30 units)	NPH and regular insulin
**Lain** **2009**	41/41	32.2/31.2	30.8/30.6	33.4/30.9	5.61–9.83/5.64–9.62	5.0/5.0	Starting dose: 2.5 mg/day; Dose titration: 2.5–5 mg increase per week as necessary; Maximum dose: 20 mg/day	Dosed at 0.8U/kg in multiple daily injections with long acting and short acting insulin and increased up to twice weekly	NA
**Mukhopadhyay** **2012**	30/30	26.3/26.0	28.3/27.4	23.7/23.0	NA	6.3/6.5	Starting dose: 2.5 mg/day; Dose titration: 2.5 mg increase weekly as necessary; Maximum dose: 20 mg/day	Starting dose: 0.7U/kg, 3 times daily; Increased weekly as necessary	NA

Abbreviations are as follows: GA, gestational age; Gly, glyburide group; Ins, insulin group; BMI, body mass index; HbA1c, glycated haemoglobin; NA, not available; U/kg, units per kilogram; OGTT, oral glucose tolerance test.

**Table 3:** Baseline characteristics of studies comparing glyburide and insulin in the treatment of GDM.

Author/year	No.of patients (Gly/Ins)	Age (years) (Gly/Ins)	GA at entry (weeks) (Gly/Ins)	Prepregnancy BMI(Kg/m2) (Gly/Ins)	OGTT fasting- 2h blood glucose (mmol/l) (Gly/Ins)	HbA1c at entry(%) (Gly/Ins)	Glyburide	Insulin	The type of insulin
**Tempe** **2013**	32/32	26.9/27.5	25.9/27.3	NA	NA	NA	Starting dose: 2.5 mg/day; Dose titration: 2.5 mg increase very 3 days as necessary; Maximum dose: 20 mg/day	Not reported; Dose was increased as necessary	NA
**Mirzamoradi** **2015**	37/59	29.50/31.18	29.9/30.3	30.18/31.77	NA	NA	Starting dose: 1.25 mg/day; Dose titration: 1.25 mg increase very 3 to 7days as necessary; Maximum dose: 20 mg/day	Starting dose: 0.4U/kg; Dose was adjusted every 2 days	NPH and regular insulin
**Behrashi** **2016**	120/129	30.69/29.98	24.89/24.48	21.94/22.59	NA	5.98/6.13	Starting dose: 1.25 mg/day; Dose titration: 1.25 to 2.5mg increase very 3 days as necessary; Maximum dose: 20 mg/day	Starting dose: 0.2U/kg; Increased every 3 days if necessary	NPH and regular insulin

Abbreviations are as follows: GA, gestational age; Gly, glyburide group; Ins, insulin group; BMI, body mass index; HbA1c, glycated haemoglobin; NA, not available; U/kg, units per kilogram; OGTT, oral glucose tolerance test.

**Table 4:** Baseline patient characteristics in studies comparing glyburide vs insulin in women with gestational diabetes mellitus.

	No. of studies	No. of patients glyburide	No. of patients insulin	Mean difference (95% CI)	P value	I^2^ value
**Maternal age (years)**	9	527	570	-0.21 (-0.86 to 0.43)	0.52	41
**Prepregnancy BMI (Kg/m**^**2**^**)**	8	342	384	-0.38 (-0.95 to 0.20)	0.20	17
**Gestational age at entry (weeks)**	9	551	592	0.45 (-0.05 to 0.96)	0.08	10
**Fasting plasma glucose at OGTT (mmol/l)**	3	290	293	-0.21 (-0.53 to 0.12)	0.21	71
**2h postprandial glucose at OGTT (mmol/l)**	3	290	293	-0.23 (-0.88 to 0.42)	0.48	65
**HbA1c at entry (%)**	6	450	465	-0.32 (-0.68 to 0.04)	0.08	88

Abbreviations are as follows: BMI, body mass index; HbA1c, glycated haemoglobin; OGTT, oral glucose tolerance test.

### Assessment of risk of bias

**Fig 2:**
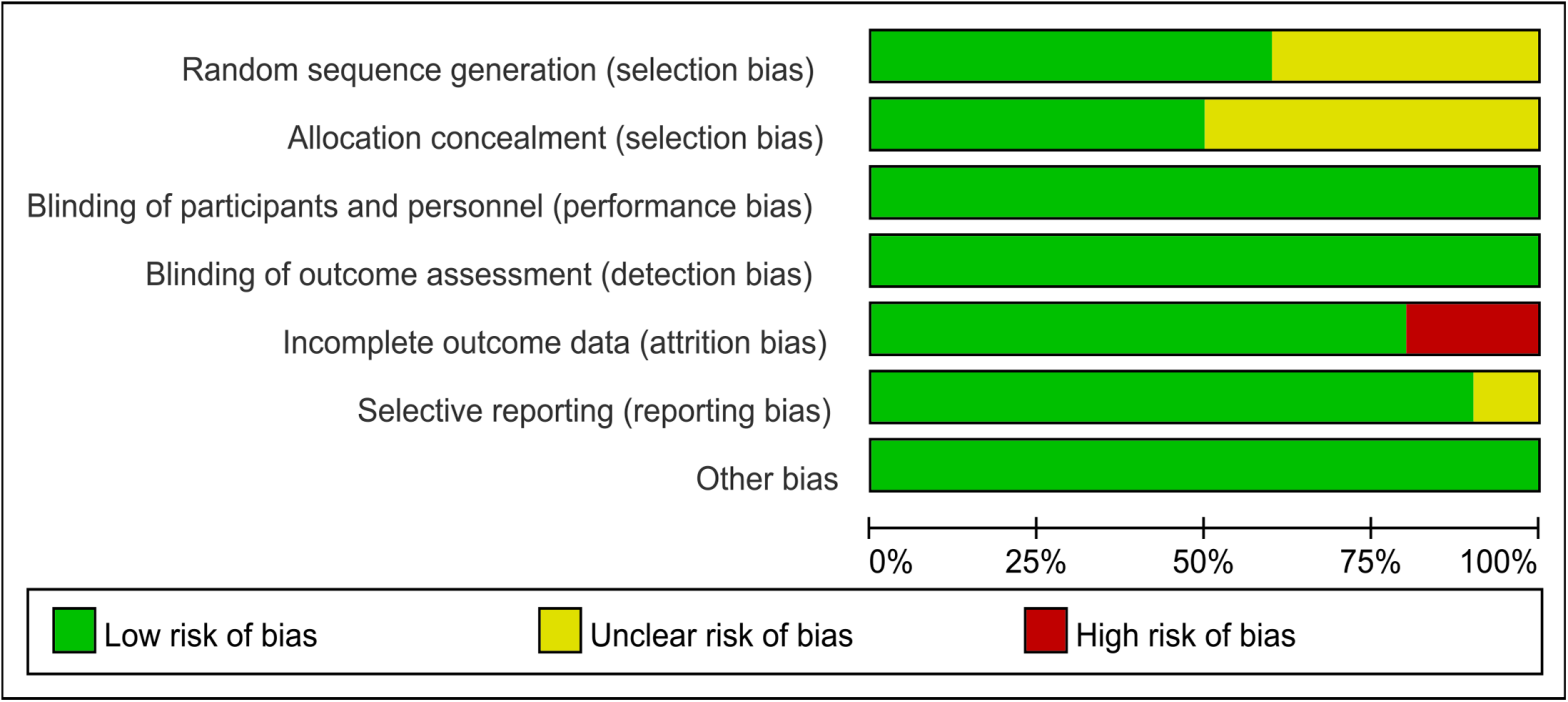
Risk of bias of studies comparing glyburide and insulin.

**Fig 3:**
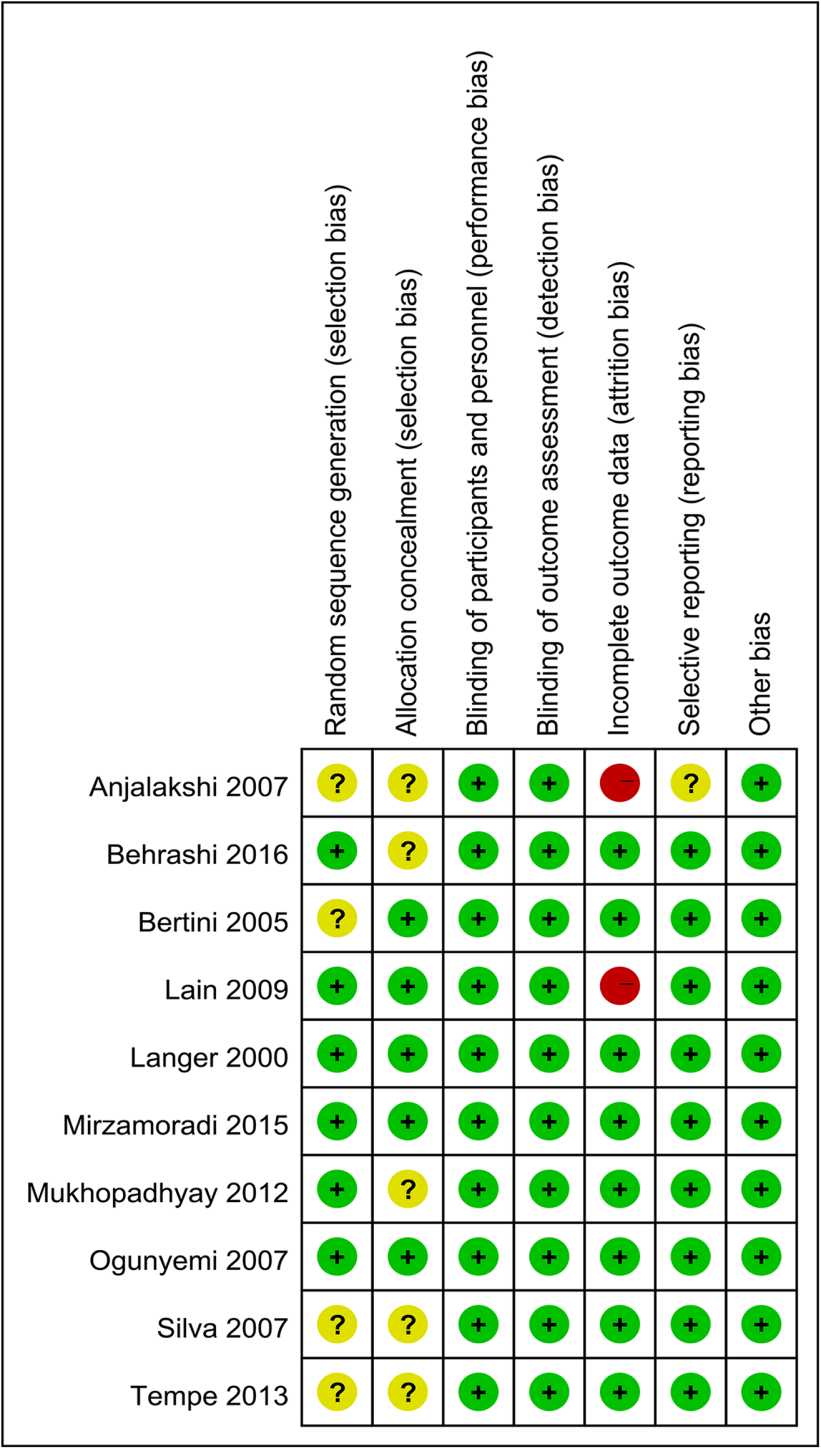
Risk of bias summary of studies comparing glyburide and insulin.

We performed quality assessments of the 10 included studies. Fig 2 illustrates the risk of bias of the included studies comparing glyburide and insulin, with the summary shown in Fig 3. The studies by Mirzamoradi [29] and Behrashi [32] are the new RCTs included in our meta-analysis. Six of the studies provided specific sequence generation methods [19, 28, 29, 32, 36, 37], while the remaining four studies did not offer information on this domain [24, 33–35]. Allocation concealment was provided in five studies [19, 29, 33, 36, 37], whereas this information was unclear in the other five studies [24, 28, 32, 34, 35]. For blinding of participants and personnel, blinding of outcomes assessment, we found low risk of bias in all the included studies. We observed attrition bias in two studies [35–37], while the other eight studies provided relatively complete outcomes data. Only one study showed unclear risk of bias for selective reporting [35], while for the rest, no selective reporting was noticed. No other risk of bias was observed in all the included studies. Most of the data in this meta-analysis was from studies at low risk of bias.

### Primary outcomes

**Fig 4:**
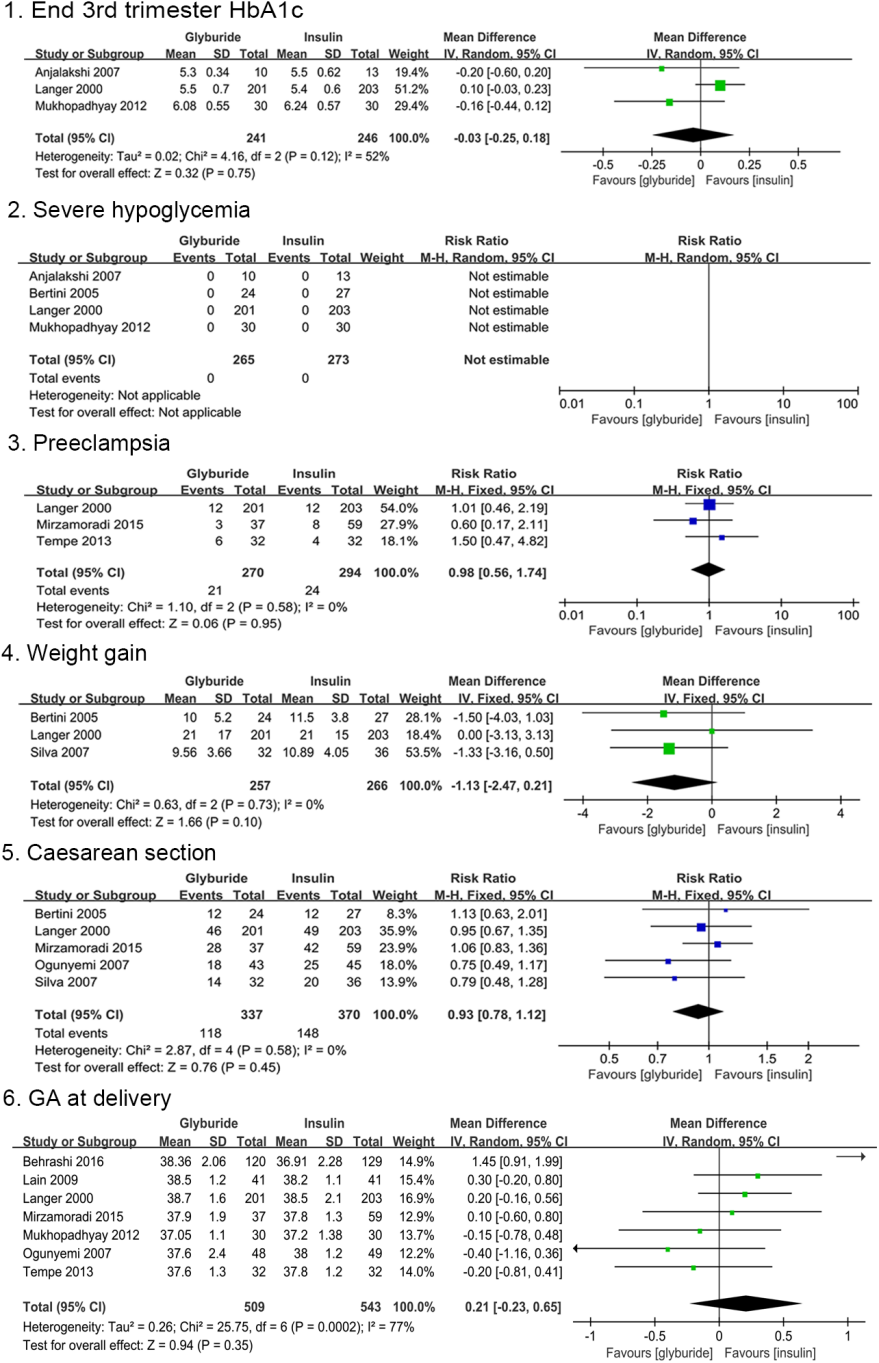
Maternal outcomes comparing glyburide and insulin.

None of the primary maternal outcomes presented a significant difference between the glyburide and insulin groups as shown in Fig 4. No significant differences were found with regard to HbA1c level at the end of third trimester [MD, -0.03; 95%CI, -0.25 to 0.18] and gestational age at delivery [MD, 0.21; 95%CI, -0.23 to 0.65] comparing glyburide and insulin. There were no cases of severe maternal hypoglycemia in the two groups available in four studies. Severe maternal hypoglycaemia was defined as ‘maternal hypoglycemia requiring hospital admission’ in the study by Bertini et al [24]. Langer et al [19] defined it as ‘blood glucose concentrations below 2.2 mmol/L, with severe symptoms’. However, the definition was not available in trials conducted by Anjalakshi et al [35] or Mukhopadhyay et al [37]. There were no significant differences in the risk of pre-eclampsia [RR, 0.98; 95%CI, 0.56 to 1.74] and caesarean section [RR, 0.93; 95%CI, 0.78 to 1.12] between the two groups. Maternal weight gain during pregnancy was provided in three trials, with insulin showing higher maternal weight gain compared to glyburide [MD, -1.13; 95%CI, -2.47 to 0.21], although, this difference was not statistically significant.

**Fig 5:**
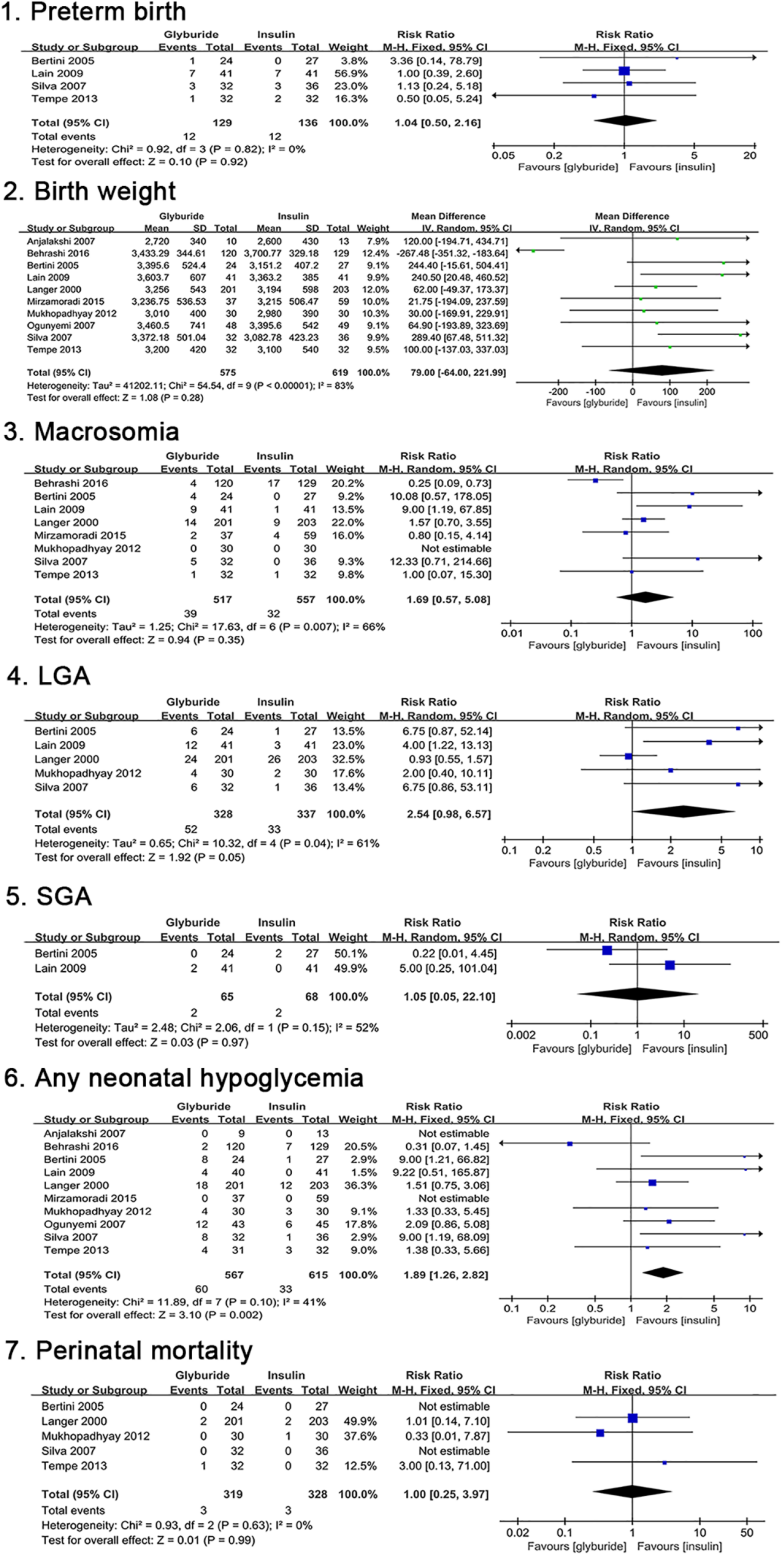
Neonatal outcomes comparing glyburide and insulin.

The risk of preterm birth did not differ between the two treatment groups [RR, 1.04; 95%CI, 0.50 to 2.16]. Birth weight appeared slightly higher in patients receiving glyburide than those receiving insulin [MD, 79; 95%CI, -64.00 to 221.99; p = 0.28], but the difference showed no statistical significance. Glyburide increased the incidence of large for gestational age (LGA) compared to insulin [RR, 2.54; 95%CI, 0.98 to 6.57; p = 0.05], however this difference did not achieve statistical significance. There were no significant differences in the risks of small for gestational age (SGA) [RR, 1.05; 95%CI, 0.05 to 22.10] and perinatal mortality [RR, 1.00; 95%CI, 0.25 to 3.97] between the two groups. Neonatal hypoglycemia was reported in ten studies. ‘Neonatal hypoglycaemia’ was consistently defined as ‘blood glucose < 40 mg/dL’ in 6 studies [19, 24, 28, 29, 32, 34]. Mukhopadhyay et al [37] indicated that ‘a cut-off of 44 mg/dL was taken to define neonatal hypoglycemia’. Additionally, the definition of ‘neonatal hypoglycaemia’ was not available in another 3 studies [33, 35, 36]. The results indicated that treatment with glyburide significantly increased the incidence of any neonatal hypoglycemia compared to treatment with insulin [RR, 1.89; 95%CI, 1.26 to 2.82; p = 0.002]. Comparisons of neonatal outcomes are shown in Fig 5. All the primary outcomes were reported as shown in Table 5.

**Table 5:** Summary of outcomes comparing glyburide with insulin in women with gestational diabetes.

Primary Outcomes		No. of studies	No. of patients treated with glyburide	No. of patients treated with insulin	Mean difference (95% CI)	Relative risk (95% CI)	P value	I^2^ value
maternal	HbA1c level at the end of third trimester (%)	3	241	246	-0.03(-0.25 to 0.18)	—	0.75	52
	Severe maternal hypoglycaemia (%)	4	265	273	—	0 v 0	—	—
	Pre-eclampsia (%)	3	270	294	—	0.98(0.56 to 1.74)	0.95	0
	Maternal weight gain during pregnancy (kg)	3	257	266	-1.13(-2.47 to 0.21)	—	0.1	0
	Caesarean section (%)	5	337	370	—	0.93(0.78 to 1.12)	0.45	0
	Gestational age at delivery (weeks)	7	509	543	0.21(-0.23 to 0.65)	—	0.35	77
neonatal	Preterm birth (%)	4	129	136	—	1.04(0.50 to 2.16)	0.92	0
	Birth weight (g)	10	575	619	79(-64.00 to 221.99)	—	0.28	83
	Macrosomia (%)	8	517	557	—	1.69(0.57 to 5.08)	0.35	66
	Large for gestational age (%)	5	328	337	—	2.54(0.98 to 6.57)	0.05	61
	Small for gestational age (%)	2	65	68	—	1.05(0.05 to 22.10)	0.97	52
	Any neonatal hypoglycaemia (%)	10	567	615	—	1.89(1.26 to 2.82)	0.002	41
	Perinatal mortality (%)	5	319	328	—	1.00(0.25 to 3.97)	0.99	0

Abbreviations are as follows: CI, confidence interval; I^2^, heterogeneity; HbA1c, glycated haemoglobin.

### Secondary outcomes

Nine secondary maternal outcomes were reported as shown in Table 6. Fasting blood glucose levels were reported in four studies. Our results indicate that insulin decreased fasting blood glucose levels compared to glyburide [MD, 1.13; 95%CI, -0.13 to 2.39; p = 0.08], however this difference was not significant. There was no statistical significance with regard to postprandial blood glucose level between the two groups [MD, 1.15; 95%CI, -2.00 to 4.31]. Maternal weight gain since entry, pregnancy-induced hypertension, induction and assisted vaginal delivery were not reported in any of the included studies. Two trials provided information on maternal trauma. There was no case in both groups. The level of Cord C peptide was reported in only one study, with no statistical significance was observed between the two groups [MD, 0.20; 95%CI, -0.42 to 0.82]. The level of Cord insulin was reported in three studies and appeared to be slightly higher in patients receiving insulin than those receiving glyburide [MD, -0.62; 95%CI, -2.93 to 1.69].

**Table 6:** Summary of outcomes comparing glyburide with insulin in women with gestational diabetes.

Secondary Outcomes		No. of studies	No. of patients treated with glyburide	No. of patients treated with insulin	Mean difference (95% CI)	Relative risk (95% CI)	P value	I^2^ value
maternal	Fasting blood glucose (mmol/L)	4	394	409	1.13(-0.13 to 2.39)	—	0.08	0
	Postprandial blood glucose (mmol/L)	3	274	280	1.15(-2.00 to 4.31)	—	0.47	0
	Maternal weight gain since entry (kg)	0	—	—	—	—	—	—
	Pregnancy induced hypertension (%)	0	—	—	—	—	—	—
	Induction (%)	0	—	—	—	—	—	—
	Maternal trauma (%)	2	73	77	—	0 v 0	—	—
	Assisted vaginal delivery (%)	0	—	—	—	—	—	—
	Cord C peptide (ng/mL)	1	31	28	0.20(-0.42 to 0.82)	—	0.53	—
	Cord insulin (IU/mL)	3	242	244	-0.62(-2.93 to 1.69)	—	0.60	0
neonatal	1 minute Apgar score <7 (%)	0	—	—	—	—	—	—
	5 minute Apgar score <7 (%)	0	—	—	—	—	—	—
	Severe neonatal hypoglycaemia (%)	5	170	222	—	4.67(0.80 to 27.22)	0.09	0
	Neonatal hyperbilirubinemia (%)	4	383	394	—	1.36(0.77 to 2.41)	0.29	0
	Phototherapy (%)	2	67	89	—	0.96(0.74 to 1.24)	0.75	29
	Neonatal respiratory distress syndrome (%)	4	292	319	—	0.73(0.32 to 1.66)	0.46	0
	Stillbirth (%)	2	233	235	—	1.68(0.22 to 12.52)	0.62	0
	Neonatal mortality (%)	3	274	280	—	1.01(0.06 to 16.04)	0.99	—
	NICU admission (%)	6	461	494	—	0.87(0.55 to 1.37)	0.54	0
	Congenital abnormality (%)	6	463	498	—	1.07(0.48 to 2.40)	0.87	0
	Hypocalcemia (%)	4	390	423	—	0.57(0.12 to 2.70)	0.48	0
	Polycythemia (%)	3	270	294	—	0.67(0.19 to 2.35)	0.54	—
	Birth trauma (%)	4	217	233	—	0 v 0	—	—
	Shoulder dystocia (%)	1	41	41	—	0.50(0.05 to 5.30)	0.57	—
	Head circumference (cm)	1	41	41	0.30(-0.31 to 0.91)	—	0.33	—
	Arm circumference (cm)	1	41	41	0.20(-0.22 to 0.62)	—	0.35	—
	Chest circumference (cm)	1	41	41	0.80(0.07 to 1.53)	—	0.03	—

Abbreviations are as follows: CI, confidence interval; I^2^, heterogeneity; NICU, neonatal intensive care unit.

Other neonatal outcomes were shown in Table 6. No study reported the incidence of 1 minute Apgar score <7 or 5 minute Apgar score <7. Five studies reported severe neonatal hypoglycemia. Severe neonatal hypoglycaemia was defined as ‘required intravenous therapy or special care’ in 3 studies [24, 34, 36]; however, the definition was not available in another 2 studies [29, 32]. Treatment with glyburide increased the incidence of severe neonatal hypoglycemia compared to treatment with insulin, but there was no statistically significant difference [RR, 4.67; 95%CI, 0.80 to 27.22; p = 0.09]. There were no significant differences in the risks of neonatal hyperbilirubinemia [RR, 1.36; 95%CI, 0.77 to 2.41], phototherapy [RR, 0.96; 95%CI, 0.74 to 1.24], neonatal respiratory distress syndrome [RR, 0.73; 95%CI, 0.32 to 1.66], stillbirth [RR, 1.68; 95%CI, 0.22 to 12.52], neonatal mortality [RR, 1.01; 95%CI, 0.06 to 16.04], NICU (neonatal intensive care unit) admission [RR, 0.87; 95%CI, 0.55 to 1.37], congenital abnormality [RR, 1.07; 95%CI, 0.48 to 2.40], hypocalcemia [RR, 0.57; 95%CI, 0.12 to 2.70], polycythemia [RR, 0.67; 95%CI, 0.19 to 2.35] or shoulder dystocia [RR, 0.50; 95%CI, 0.05 to 5.30] between the two groups. Information on birth trauma was reported in four studies, there was no case of birth trauma in the two groups. Only one study reported head, arm and chest circumferences, with no statistical significant difference observed between the two groups with regard to head [MD, 0.30; 95%CI, -0.31 to 0.91] and arm [MD, 0.20; 95%CI, -0.22 to 0.62] circumference, whereas glyburide significantly increased chest circumference compared to insulin [MD, 0.80; 95%CI, 0.07 to 1.53; p = 0.03].

### Heterogeneity test and sensitivity analysis

Sources of heterogeneity were detected as statistically significant heterogeneity existing in several primary outcomes, such as gestational age at delivery, birth weight, macrosomia and any neonatal hypoglycemia. Funnel plots indicated that the newest study, published in 2016 [32] was the major source for heterogeneity. Sensitivity analysis was performed for the above primary outcomes after excluding the newest study. As shown in S1 Fig, no difference was observed for gestational age at delivery [MD, 0.06; 95%CI, -0.16 to 0.28], which is consistent with the previous result, but I^2^ decreased from 77% to 0%. After excluding the newest study, birth weight appeared higher in patients receiving glyburide than in those receiving insulin [MD, 109.16; 95%Cl, 42.59 to 175.72; p = 0.001], with I^2^ decreasing from 83% to 0%. Risk of macrosomia appeared higher in patients receiving glyburide than in those receiving insulin [RR, 2.48; 95%CI, 1.38 to 4.44; p = 0.002], with I^2^ decreasing from 66% to 30%. Risk for any neonatal hypoglycemia still remained significantly higher with glyburide compared to insulin [RR, 2.29; 95%CI, 1.49 to 3.54; p = 0.0002], with I^2^ decreased from 41% to 13%. There was no significant heterogeneity for the remaining outcomes. S2 Table shows the summary outcomes in the sensitivity analysis.

## Discussion

Recently, oral hypoglycemic agents have been identified as alternatives to insulin in the management of GDM. An alternative was sought owing to high insulin costs, inconvenience, and the probability of a higher risk of hypoglycemia. This updated meta-analysis was conducted to evaluate the efficacy and safety of glyburide in patients with GDM and to compare it with insulin therapy. Several maternal and neonatal outcomes were assessed.

Maternal glycemic control was not significantly different between the two treatment groups in our meta-analysis, which indicated that glyburide and insulin were equally effective for the treatment of GDM. This was consistent with previous studies [26–28, 30]. However, with respect to the oral treatment of GDM, metformin may be preferred over glyburide as first-line therapy, according to a recent RCT conducted by Nachum Z et al [38]. In addition, similar to the results reported by Balsells et al [27], there were no cases of severe maternal hypoglycemia reported in the 10 trials included in this meta-analysis. Furthermore, there were no differences in the other primary indicators of maternal outcome, including weight gain, pre-eclampsia, caesarean section, and gestational age at delivery, between the two groups, which was also consistent with the findings of previous studies [27, 39]. However, Malek et al [40] found that the risk of pre-eclampsia was higher in the group treated with glyburide than in the group treated with insulin. With respect to secondary maternal outcomes, such as maternal trauma, Cord C peptide, and Cord insulin, our results were consistent with those of a previous review [27], which suggested that glyburide did not harm the mother compared to insulin.

Neonatal hypoglycemia frequently occurs in infants of women with GDM. All trials included in this meta-analysis reported the incidence of neonatal hypoglycemia and the results indicated a higher risk of any neonatal hypoglycemia after maternal treatment with glyburide compared to treatment with insulin (p = 0.002). This was consistent with several previous meta-analyses [26, 27, 39, 40] and a sensitivity analysis confirmed the robustness of this result. Like any neonatal hypoglycemia, treatment with glyburide resulted in an increase in the incidence of severe neonatal hypoglycemia compared to treatment with insulin; however, the difference was not statistically significant.

All trials included in this meta-analysis indicated that maternal treatment with glyburide resulted in an increased birth weight compared to treatment with insulin, except the newest large-scale RCT carried out by Behrashi et al [32]. Our results suggested that there was no difference between the two groups with regard to birth weight, which was different from a previous conclusion [27]. Macrosomia was consistently defined as birth weight no less than 4000 g in the 6 evaluated studies. Like birth weight, no differences were observed between the two groups with respect to the risk of macrosomia in our meta-analysis. Similarly, several studies showed no significant difference between the insulin and glyburide groups in the prevalence of macrosomia [1, 41, 42]. However, several other studies [27, 30, 40, 43] indicated that glyburide was associated with a higher incidence of macrosomia. Conversely, Behrashi et al [32] found that the incidence of macrosomia in the glyburide group was significantly lower than that in the group that received insulin. This new trial [32] was the largest trial in recent years. However, the time when birth weight was measured was not standard and the starting insulin dose was 0.2 U/kg, which was lower than that used in other trials. It is possible that these two factors accounted for the different results in this trial. A heterogeneity test was conducted by excluding the newest study and the findings indicated that birth weight was significantly higher when the mothers received glyburide than when the mothers received insulin. In addition, macrosomia occurred significantly more often when the mothers received glyburide than when the mothers received insulin.

In this meta-analysis, there was no statistically significant difference between the treatment groups with respect to the incidence of neonatal hyperbilirubinemia and phototherapy. Balsells et al [27] also found no difference in the incidence of neonatal jaundice between the two treatment groups. Other secondary neonatal outcomes, including neonatal respiratory distress syndrome, stillbirth, and NICU admission, were not significantly different between the two groups. This was also similar to the conclusion of Balsells et al [27]. However, the results of Malek et al [40] indicated that the risk of NICU admission was higher in the group treated with glyburide than in the group treated with insulin. In addition, our results indicated that there were no significant differences in the prevalence of hypocalcemia, polycythemia, birth trauma, shoulder dystocia, or neonatal circumference between the two groups. These indicators were all evaluated for the first time in our study when compared to other studies [27, 40].

In summary, the use of glyburide in pregnancy for women with GDM appears to be as effective as the use of insulin, but neonatal hypoglycemia should be monitored. Additionally, the potential risk of glyburide to the fetus is unclear, especially over the long-term, and should be reassessed in the future because the evidence that indicates glyburide is noticeable in the fetal circulation [44].

### Limitations in current evidence

There were several limitations in our present meta-analysis that deserve comment. First, only one original study that was not written in English was included in this meta-analysis, which could have resulted in bias or limited our ability to draw substantial conclusions. Second, some outcomes were reported in only one study or no cases were reported in some of the trials included in the meta-analysis, which limited the analysis of some of the outcomes of interest. Third, none of these studies evaluated long-term maternal and neonatal outcomes. In the future, additional long-term data on maternal and neonatal outcomes should be evaluated to confirm the safety of glyburide use in women with GDM. Moreover, the quality assessment of the included trials indicated that not all of the studies were of high quality.

## Conclusions

Our study indicated that glyburide was safe and effective for use in GDM, provided neonates are monitored for hypoglycemia.

## Supporting information

S1 Table Search strategy. (PDF)

S2 Table Summary of meta-analysis outcomes comparing glyburide with insulin in women with gestational diabetes.Sensitivity analysis. (PDF)

S3 Table PRISMA checklist. (PDF)

S1 Fig Maternal and neonatal outcomes comparing glyburide and insulin (sensitivity analysis). (TIF)

## Abbreviations

GDM: gestational diabetes mellitus
GA: gestational age
LGA: large for gestational age
SGA: small for gestational age
BMI: body mass index
HbA1c: glycated haemoglobin
OGTT: oral glucose tolerance test
NICU: neonatal intensive care unit
95%CI: 95% confidence interval
I^2^: heterogeneity
