# Supplementary material for: Comparison of glyburide and insulin in the management of gestational diabetes: A meta-analysis
Source: PLoS One. 2017 Aug 3;12(8):e0182488. doi: 10.1371/journal.pone.0182488 (PMC5542468; doi:10.1371/journal.pone.0182488)
Supplement: S1 Table — (PDF) [file pone.0182488.s001.pdf]

S1 Table. Search strategy.

PubMed

#1	“Diabetes, Gestational”[MeSH]	8720
#2	Diabetes, Pregnancy-Induced[tiab]	0
#3	Diabetes, Pregnancy Induced[tiab]	0
#4	Pregnancy-Induced Diabetes[tiab]	10
#5	Gestational Diabetes[tiab]	9903
#6	Diabetes Mellitus, Gestational[tiab]	1
#7	Gestational Diabetes Mellitus[tiab]	5253
#8	#1 or #2 or #3 or #4 or #5 or #6 or #7	12739
#9	“Glyburide”[MeSH]	5811
#10	Glybenclamide[tiab]	447
#11	Glibenclamide[tiab]	7483
#12	Diabeta[tiab]	8
#13	Euglucon 5[tiab]	6
#14	Neogluconin[tiab]	1
#15	HB-419[tiab]	88
#16	HB 419[tiab]	88
#17	HB419[tiab]	8
#18	HB-420[tiab]	7
#19	HB 420[tiab]	7
#20	HB420[tiab]	1
#21	Maninil[tiab]	15
#22	Micronase[tiab]	4
#23	Daonil[tiab]	44
#24	Euglucon N[tiab]	6
#25	#9 or #10 or #11 or #12 or #13 or #14 or #15 or #16 or #17 or #18 or #19 or #20 or #21 or #22 or #23 or #24	9760
#26	randomized controlled trial	536766
#27	controlled clinical trial	613273
#28	randomized	727135
#29	placebo	193825
#30	drug therapy	2706504
#31	randomly	259820
#32	trial	1152022
#33	groups	1729302
#34	#26 or #27 or #28 or #29 or #30 or #31 or #32 or #33	4837848
#35	#8 and #25 and #34	121

EMBASE

#1 ‘Pregnancy diabetes mellitus’/exp 25846
#2 ‘Diabetes, Gestational’:ab,ti 201
#3 ‘Diabetes, Pregnancy-Induced’:ab,ti 34
#4 ‘Diabetes, Pregnancy Induced’:ab,ti 34
#5 ‘Pregnancy-Induced Diabetes’:ab,ti 9
#6 ‘Gestational Diabetes’:ab,ti 15032
#7 ‘Diabetes Mellitus, Gestational’:ab,ti 58
#8 ‘Gestational Diabetes Mellitus’:ab,ti 7139
#9 #1 or #2 or #3 or #4 or #5 or #6 or #7 or #8 27146
#10 ‘Glibenclamide’/exp 21997
#11 ‘Glybenclamide’:ab,ti 564
#12 ‘Glyburide’:ab,ti 1854
#13 ‘Diabeta’:ab,ti 688
#14 ‘Euglucon 5’:ab,ti 10
#15 ‘Neogluconin’:ab,ti 1
#16 ‘HB-419’:ab,ti 95
#17 ‘HB 419’:ab,ti 95
#18 ‘HB419’:ab,ti 9
#19 ‘HB-420’:ab,ti 10
#20 ‘HB 420’:ab,ti 10
#21 ‘HB420’:ab,ti 1
#22 ‘Maninil’:ab,ti 19
#23 ‘Micronase’:ab,ti 28
#24 ‘Daonil’:ab,ti 72
#25 ‘Euglucon N’:ab,ti 10
#26 #10 or #11 or #12 or #13 or #14 or #15 or #16 or #17 or #18 or #19 or #20 or #21 or #22 or #23 or #24 or #25 23015
#27 ‘Randomized controlled trial’/exp 415659
#28 #9 and #26 and #27 26

CENTRAL (Cochrane Central Register of Controlled Trials)

#1	MeSH descriptor: [Diabetes, Gestational] explode all trees	479
#2	Diabetes, Pregnancy-Induced:ti,ab	47
#3	Diabetes, Pregnancy Induced:ti,ab	99
#4	Pregnancy-Induced Diabetes:ti,ab	47
#5	Gestational Diabetes:ti,ab	1086
#6	Diabetes Mellitus, Gestational:ti,ab	829
#7	Gestational Diabetes Mellitus:ti,ab	829
#8	Pregnancy diabetes mellitus:ti,ab	1024
#9	#1 or #2 or #3 or #4 or #5 or #6 or #7 or #8	1385
#10	MeSH descriptor: [Glyburide] explode all trees	507
#11	Glybenclamide:ti,ab	6
#12	Glibenclamide:ti,ab	783
#13	Diabeta:ti,ab	2
#14	Euglucon 5:ti,ab	9
#15	Neogluconin:ti,ab	1
#16	HB-419:ti,ab	8
#17	HB 419:ti,ab	11
#18	HB419:ti,ab	5
#19	HB-420:ti,ab	8
#20	HB 420:ti,ab	17
#21	HB420:ti,ab	1
#22	Maninil:ti,ab	3
#23	Micronase:ti,ab	5
#24	Daonil:ti,ab	13
#25	Euglucon N:ti,ab	9
#26	#10 or #11 or #12 or #13 or #14 or #15 or #16 or #17 or #18 or #19 or #20 or #21 or #22 or #23 or #24 or #25	980
#27	#9 and #26	30 (27 Trials; 3 Other Reviews)
