# Supplementary material for: Comparison of glyburide and insulin in the management of gestational diabetes: A meta-analysis
Source: PLoS One. 2017 Aug 3;12(8):e0182488. doi: 10.1371/journal.pone.0182488 (PMC5542468; doi:10.1371/journal.pone.0182488)
Supplement: S2 Table — (PDF) [file pone.0182488.s002.pdf]

S2 Table. Summary of meta-analysis outcomes comparing glyburide with insulin in women with gestational diabetes. Sensitivity analysis.

Outcomes			No. of studies	No. of patients treated with glyburide	No. of patients treated with insulin	Mean difference (95% Cl)	Relative risk (95% Cl)	P value	I^2 value
Primary outcomes	Maternal outcomes	HbA1c level at end of third trimester (%)	3	241	246	-0.03(-0.25 to 0.18)	—	0.75	52
		Severe maternal hypoglycaemia (%)	4	265	273	—	0 v 0	—	—
		Pre-eclampsia (%)	3	270	294	—	0.98(0.56 to 1.74)	0.95	0
		Maternal weight gain during pregnancy (kg)	3	257	266	-1.13(-2.47 to 0.21)	—	0.1	0
		Caesarean section (%)	5	337	370	—	0.93(0.78 to 1.12)	0.45	0
		Gestational age at delivery (weeks)	6	389	414	0.06(-0.16 to 0.28)	—	0.57	0
	Neonatal outcomes	Preterm birth (%)	4	129	136	—	1.04(0.50 to 2.16)	0.92	0
		Birth weight (g)	9	455	490	109.16(42.59 to 175.72)	—	0.001	0
		Macrosomia (%)	7	397	428	—	2.48(1.38 to 4.44)	0.002	30
		Large for gestational age (%)	5	328	337	—	2.54(0.98 to 6.57)	0.05	61
		Small for gestational age (%)	2	65	68	—	1.05(0.05 to 22.10)	0.97	52
		Any neonatal hypoglycaemia (%)	9	447	486	—	2.29(1.49 to 3.54)	0.0002	13
		Perinatal mortality (%)	5	319	328	—	1.00(0.25 to 3.97)	0.99	0
Secondary outcomes	Maternal outcomes	Fasting blood glucose (mmol/L)	3	274	280	1.19(-1.12 to 3.50)	—	0.31	0
		Postprandial blood glucose (mmol/L)	3	274	280	1.15(-2.00 to 4.31)	—	0.47	0
		Maternal weight gain since entry (kg)	0	—	—	—	—	—	—
		Pregnancy induced hypertension (%)	0	—	—	—	—	—	—
		Induction (%)	0	—	—	—	—	—	—
		Maternal trauma (%)	2	73	77	—	0 v 0	—	—
		Assisted vaginal delivery (%)	0	—	—	—	—	—	—
		Cord C peptide (ng/mL)	1	31	28	0.20(-0.42 to 0.82)	—	0.53	—
		Cord insulin (IU/mL)	3	242	244	-0.62(-2.93 to 1.69)	—	0.60	0
	Neonatal outcomes	1 minute Apgar score <7 (%)	0	—	—	—	—	—	—
		5 minute Apgar score <7 (%)	0	—	—	—	—	—	—
		Severe neonatal hypoglycemia (%)	4	133	163	—	4.67(0.80 to 27.22)	0.09	0
		Neonatal hyperbilirubinemia (%)	3	263	265	—	1.72(0.75 to 3.94)	0.20	0
		Phototherapy (%)	2	67	89	—	0.96(0.74 to 1.24)	0.75	29
		Neonatal respiratory distress syndrome (%)	4	292	319	—	0.73(0.32 to 1.66)	0.46	0

S2 Table. Summary of meta-analysis outcomes comparing glyburide with insulin in women with gestational diabetes. Sensitivity analysis (Continued).

Outcomes			No. of studies	No. of patients treated with glyburide	No. of patients treated with insulin	Mean difference (95% Cl)	Relative risk (95% Cl)	P value	I^2 value
Secondary outcomes	Neonatal outcomes	Stillbirth (%)	2	233	235	—	1.68(0.22 to 12.52)	0.62	0
		Neonatal mortality (%)	3	274	280	—	1.01(0.06 to 16.04)	0.99	—
		NICU admission (%)	5	341	365	—	0.95(0.58 to 1.57)	0.85	0
		Congenital abnormality (%)	5	343	369	—	1.31(0.55 to 3.13)	0.54	0
		Hypocalcemia (%)	3	270	294	—	1.01(0.14 to 7.10)	0.99	—
		Polycythemia (%)	3	270	294	—	0.67(0.19 to 2.35)	0.54	—
		Birth trauma (%)	3	97	104	—	0 v 0	—	—
		Shoulder dystocia (%)	1	41	41	—	0.50(0.05 to 5.30)	0.57	—
		Head circumference (cm)	1	41	41	0.30(-0.31 to 0.91)	—	0.33	—
		Arm circumference (cm)	1	41	41	0.20(-0.22 to 0.62)	—	0.35	—
		Chest circumference (cm)	1	41	41	0.80(0.07 to 1.53)	—	0.03	—

Abbreviations are as follows: Cl, confidence interval; I^2, heterogeneity; HbA1c, glycated haemoglobin; NICU, neonatal intensive care unit.
