# Supplementary figures and images for: Comparison of glyburide and insulin in the management of gestational diabetes: A meta-analysis
Source: PLoS One. 2017 Aug 3;12(8):e0182488. doi: 10.1371/journal.pone.0182488 (PMC5542468; doi:10.1371/journal.pone.0182488)

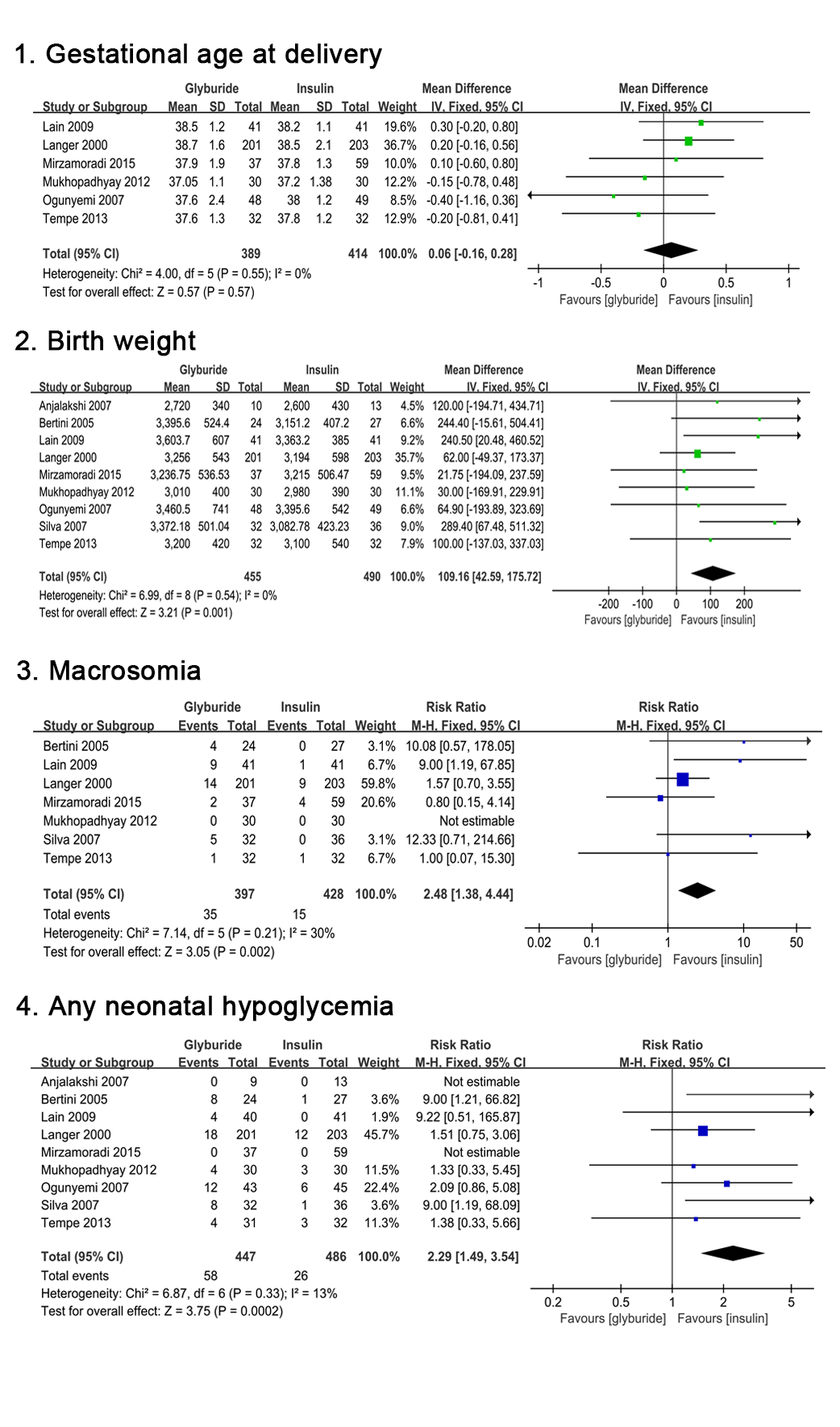

Supplement: S1 Fig — (TIF) [file pone.0182488.s004.tif]
